# Nanostructure-Directed Chemical Sensing: The IHSAB Principle and the Effect of Nitrogen and Sulfur Functionalization on Metal Oxide Decorated Interface Response

**DOI:** 10.3390/nano3030469

**Published:** 2013-08-07

**Authors:** William I. Laminack, James L. Gole

**Affiliations:** 1 Department of Physics, Georgia Institute of Technology, Atlanta, GA 30332, USA; E-Mail: wlaminack3@gatech.edu; 2 Department of Mechanical Engineering, Georgia Institute of Technology, Atlanta, GA 30332, USA

**Keywords:** nanostructure directed sensing, nitrogen and sulfur functionalization

## Abstract

The response matrix, as metal oxide nanostructure decorated *n*-type semiconductor interfaces are modified *in situ* through direct amination and through treatment with organic sulfides and thiols, is demonstrated. Nanostructured TiO_2_, SnO*_x_*, NiO and Cu*_x_*O (*x* = 1,2), in order of decreasing Lewis acidity, are deposited to a porous silicon interface to direct a dominant electron transduction process for reversible chemical sensing in the absence of significant chemical bond formation. The metal oxide sensing sites can be modified to decrease their Lewis acidity in a process appearing to substitute nitrogen or sulfur, providing a weak interaction to form the oxynitrides and oxysulfides. Treatment with triethylamine and diethyl sulfide decreases the Lewis acidity of the metal oxide sites. Treatment with acidic ethane thiol modifies the sensor response in an opposite sense, suggesting that there are thiol (SH) groups present on the surface that provide a Brønsted acidity to the surface. The *in situ* modification of the metal oxides deposited to the interface changes the reversible interaction with the analytes, NH_3_ and NO. The observed change for either the more basic oxynitrides or oxysulfides or the apparent Brønsted acid sites produced from the interaction of the thiols do not represent a simple increase in surface basicity or acidity, but appear to involve a change in molecular electronic structure, which is well explained using the recently developed inverse hard and soft acids and bases (IHSAB) model.

## 1. Introduction

There is a substantial need to develop new materials that allow the sensing of chemicals in a broad range of environments. A combination of uniquely defined active interfaces and the ability to confine processes at the nanoscale, coupled with the ability to manipulate nanostructured materials and their interactions at select interfaces, offers a special opportunity to develop economically viable, energy-efficient and sensitive modes of detection for chemical species [1,2,3]. The ability to manipulate and control charge transport at porous semiconductor micro/nanoporous interfaces, driven by nanostructure-focused Brønsted and Lewis acid-base chemistry, can play a major role in the development of highly responsive, sensitive (ppb), reversible sensors [4,5,6]. Within this framework, the creation of novel, highly active, micro-/nano-structured porous *extrinsic* semiconductor interfaces, their ability to provide readily accessible significant light harvesting surface areas [7] and their ability to be transformed with select nanostructure interactions [1,2,3] provide new avenues for sensing based on energy transfer and transduction [8]. Nanopore coated, microporous arrays not only enable enhanced Fickian diffusion [9] to active sites, but also, the nanopores provide a “phase matching” region with which modifying nanostructured materials can be made to interact in a controlled manner to promote a distinct and controllable, wide ranging and variable interface sensitivity [10].

We have recently developed a new concept, inverse hard and soft acids and bases (IHSAB) [1,2,3], that expands on the tenants of the HSAB [11] principle and allows the design of novel sensors, as well as catalytic sites. The IHSAB principle incorporates the coupling of analyte/interface acid-base chemistry, an approach to the balance and separation of surface physisorption (electron transduction) and chemisorption, and the ability of active nanostructures to utilize these differences. Here, the concept of electron transduction [1,2,3,8] is defined as the transfer of electrons to or from an interface without the formation of a chemical bond. At the heart of the concept, based on our experimental observations, is the effective transfer of electrons to acidic or from basic molecules (analytes) at a nanostructure modified semiconductor interface. As primarily acidic metal oxides, the nanostructures focus the interaction and coupling with the majority charge carrier concentration of an extrinsic *p*- or *n*-type semiconductor, directing an electron transduction process. We have now found that these metal oxide nanostructures can be readily functionalized, *in situ*, to create what appear to be metal oxynitride [10,12] and oxysulfide [13] sites. The semiconductor interfaces can also be modified, as they are treated with nanostructured photocatalysts to provide a light-enhanced sensing efficiency. In concert, these concepts can be used to formulate solar pumped sensors [14]. In this manuscript, we outline the nature of the formation of nanostructure-modified reversible sensor interfaces and the nature of those changes that occur as these interfaces are modified *in situ* to produce oxynitride and oxysulfide sites. Our observations suggest that the observed changes can be explained within the recently developed IHSAB principle [1,2,3].

The fractional deposition of nanostructured metal oxide centers can be used to create inexpensive, micro-fabricated interfaces and selective interfacial platforms that can be developed for applications of focused electron transduction built on our IHSAB model [1,2,3]. Nanostructured metal oxide treatments modify the interface activity to create a dominance of physisorption/weak chemisorption *verses* significant chemical bonding, facilitating a reversible porous silicon (PS) gas sensor response. In order to explain this behavior, we have developed a complementary concept to that formulated by Pearson *et al.* [11,12,13,14,15] for hard and soft acid-base (HSAB) interactions. In the HSAB concept, the interaction strength is correlated with the relative acidity and basicity of the reactants, as strong acids react with strong bases and weak acids interact with weak bases, resulting in significant ionic and covalent bonding, respectively. We wish to minimize this bond formation. A nanostructure-treated PS gas sensor can be made to behave in a physisorption/weak chemisorption dominated mode, as the IHSAB concept can be used to explain this behavior [1,2,3]. Here, the physisorption process is found to dominate for primarily strong acid-weak base and weak acid-strong base interactions. The emphasis is to impede bond formation by creating a molecular orbital mismatch. By assessing trends within the IHSAB framework, appropriate selections can be made for the modification of the porous Si hybrid interface with nanostructured metal/metal oxide deposits to create a range of sensitivities for a number of gases [1,2,3,4,5,6].

**Figure 1:**
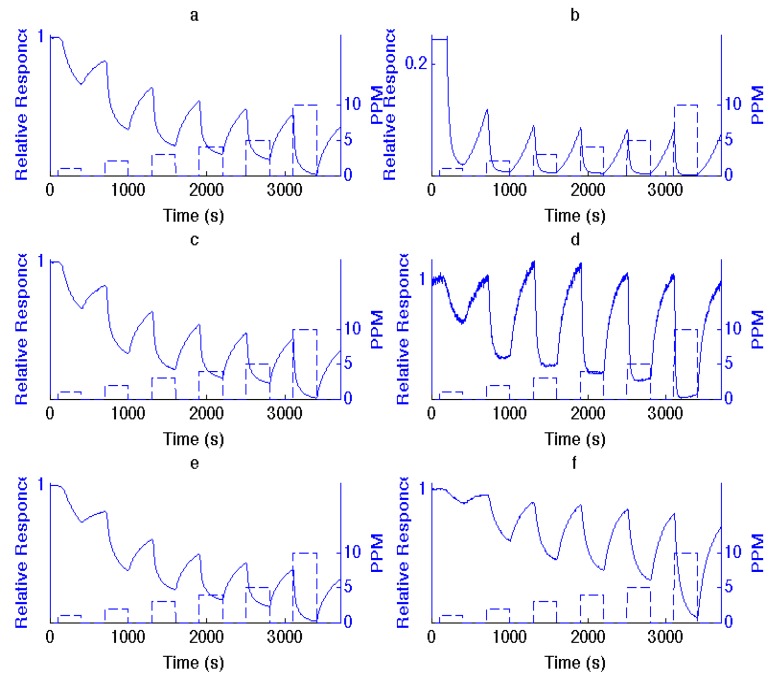
Comparison of responses to 1, 2, 3, 4, 5 and 10 ppm NH_3_ for (**a**), (**c**) and (**e**), sensors, consisting of an untreated *n*-type porous silicon (PS) interface with those treated with (**b**) TiO_2_, (**d**) SnO*_x_* and (**f**) NiO fractional nanostructured island depositions. The PS interface in (a) is that treated with TiO_2_ in (b) and similarly for SnO*_x_* and NiO.

**Figure 2:**
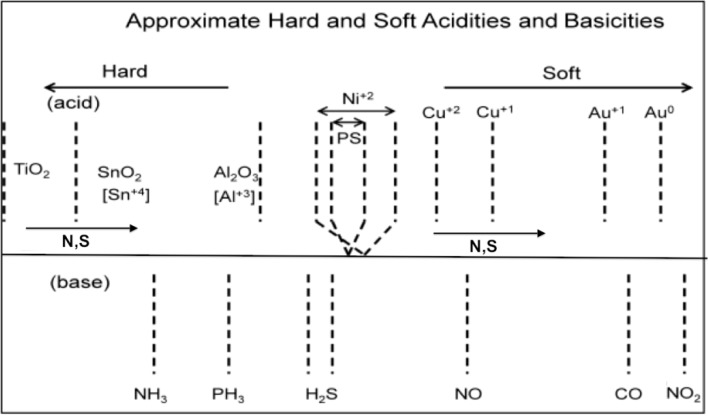
Estimated hard and soft acidities and basicities based on resistance change relative to a *p*- and *n*-type porous silicon interface. The acidic metal oxides that decorate the semiconductor interface can be modified through *in situ* nitridation and sulfurization, decreasing their Lewis acidity. The analytes remain as positioned. A horizontal line is used to separate the metal oxides used to modify the interface (above) and the analytes below in the figure.

The deposition of metal oxide nanostructures introduces new selective sites (see Figure S1), which modify the PS interface on which they are deposited. This produces an enhanced and variable response relative to an untreated interface, in direct relation to the acid strength of the deposited metal oxide, the degree of basicity or acidity of the analyte and the nature of the extrinsic semiconductor doping [1,2,3,4,5,6]. From Table S1, it is possible to infer the reversible interaction of NH_3_ with a variety of surfaces using the IHSAB model. We exemplify this interaction for select TiO_2_-, SnO*_x_*-, and NiO-treated PS interfaces in Figure 1. NH_3_, as a strong base, contributes electrons to a PS interface. For *n*-type PS, this results in an increase in the number of excess charge carriers, which are electrons, and, as Figure 1 demonstrates, a decrease in resistance (increase in conductance). Titanium oxide and tin oxide nanostructure deposits, as strong acids, significantly enhance the extraction of electrons and the response of the PS interface to NH_3_. In fact, by comparison, the responses saturate the conductance for NH_3_ concentrations greater than 2 ppm. As a stronger acid, TiO_2_ is more effective. NiO, which represents an intermediate acid, also enhances the interface response, but to a lesser degree. The changes in response depicted in Figure 1 are predictable from the IHSAB concept. By evaluating the reversible interaction of a given analyte with the nanostructure-deposited metal oxides, it is possible to construct the acid/base interaction diagram depicted in Figure 2. It is also feasible to expand the range of interface acidity by modifying the metal oxide nanostructure deposits, and we have obtained initial evidence for the facile *in situ* transformation of the metal oxides to their corresponding oxynitrides and oxysulfides [16,17] at the nanoscale. The systems that we have studied are summarized in Table 1.

**Table 1:** Summary of metal oxide nanostructure deposits and chemical functionalization using nitrogen and sulfur compounds. The systems studied and *in situ* exposures are given in the table. * signifies the change in response to the analyte, NH_3_ as summarized in the paper. # signifies the change in response with the analytes, NH_3_ and NO, shown in the paper.

Metal Oxide/Chemical Dopant	TiO_2_	SnO_2_	NiO	Cu_x_O
Triethylamine, 30 s	*	*	*	#
Thiol, 30 s		*	*	
Diethyl Sulfide, 15 s	*	*	*	

## 2. Results and Discussion

In Figure 2, the analyte scale is fixed in terms of acid/base properties, as determined by the energy of the lone pair (lone electron) donating to the positive metal site. The analyte lone pair energies can be evaluated from their ionization potentials or proton affinities (gas phase basicity). The sensor scale in Figure 2 can be varied by substituting N or S for oxygen, as they donate electron density into the metal. There is the apparent ability for *in situ* transformation of the deposited metal oxide nanostructures, which can enhance the array of distinct responses that can be developed and extended to form “*materials sensitivity matrices*” for a given analyte, as it provides a route to decrease the Lewis acidity of these acidic sites. The degree of nitridation can be used to introduce a progressively increasing site basicity at the nanoscale [16]. This transformation is easily accomplished through direct amination in a manner analogous to that applied to the facile conversion of TiO_2_ to TiO_2__−__*x*_N*_x_* [18,19,20]. The *in situ* formation of the oxynitrides shifts the transformed oxides toward the soft acid side of Figure 2, adding breadth to this material’s selectivity table.

**Figure 3:**
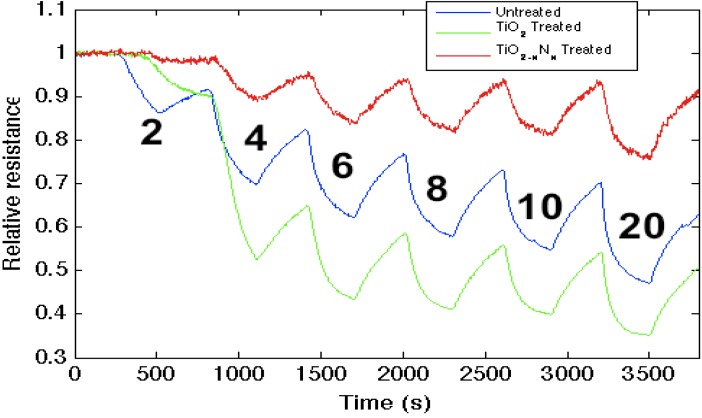
Response corresponding to decreasing resistance as NH_3_ contributes electrons to an untreated porous silicon (PS)-, TiO_2_- and TiO_2 __−__*x*_N*_x_*-treated PS interfaces. The TiO_2__−__*x*_N*_x_*-treated interface is basic relative to the PS- and TiO_2_- treated PS acidic sites.

Figure 3 demonstrates that while the strong (hard) acid, TiO_2_, increases the sensitivity of the untreated *n*-type PS interface to NH_3_, the oxynitride, TiO_2__ −__*x*_N*_x_*, formed through *in situ* amination of the TiO_2_-deposited surface, decreases the response to NH_3_. This result is consistent with the observed effect of *in situ* nitridation, as it modifies the response of the sensor interface within the IHSAB format. TiO_2_, as a strong acid, enhances the capture of electrons, transferring these electrons to increase conductance (decrease resistance) relative to the undecorated interface. The formation of the oxynitride decreases the metal site Lewis acidity and does not facilitate electron transduction as efficiently. The sensor response corresponds to a conductance decrease relative to the TiO_2_-treated interface. The *in situ* nitridation of TiO_2_ shifts the nature of this metal oxide nanostructure toward the soft acid side of Figure 2, closer to ammonia. The IHSAB principle dictates that the response of the TiO_2__ −__*x*_N*_x_* interface should decrease relative to TiO_2_, as it indeed does. However, the nitridation process does not simply increase the basicity of the nanostructure surfaces. The control of the interaction with the molecule to be sensed is dictated by orbital orientation and steric effects. A weak interaction with minimal chemical bonding occurs if the donor orbital (highest occupied molecular orbital, HOMO) energy is not well matched with the acceptor (lowest occupied molecular orbital, LUMO) energy. As the HOMO (donor)-LUMO (acceptor) energy gap decreases, there will be more charge transfer and a stronger Lewis acid-base interaction. The IHSAB principle dictates that the orbital matchup and Lewis acid-base bonding with NH_3_ is enhanced. By comparison, the sensor response of the TiO_2__ −__*x*_N*_x_* interface decreases relative to TiO_2_. In both concert and contrast to the behavior expected for a simple basic interface, the sensitivity of the weaker metal oxides can be enhanced by nitridation.

**Figure 4:**
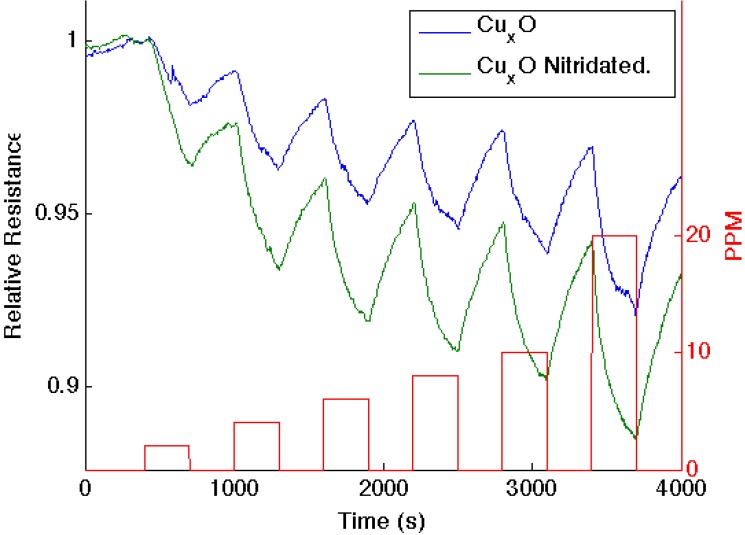
Response corresponding to decreasing resistance as NH_3_ contributes electrons to a Cu*_x_*O-treated porous silicon (**blue line**) and nitridated Cu*_x_*O nanostructure-treated PS (**green line**). The nitridated Cu*_x_*O-treated interface is basic relative to the PS- and Cu*_x_*O-treated PS acidic sites.

**Figure 5:**
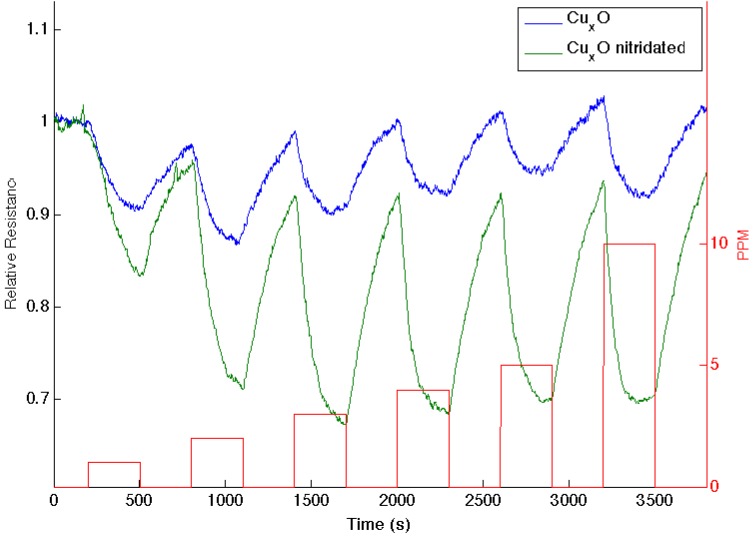
Response of a Cu*_x_*O-treated PS interface to NO (**blue line**) and after nitridation with triethylamine (**green line**). The boxes (**red line**) denote the analyte concentration from 1 to 10 ppm.

Similar decreases in the observed sensor response are observed as nitridated SnO*_x_*, whose Lewis acidity has decreased, interacts with NH_3_ and NO, where the molecular orbital makeup is now more closely aligned [16]. Figure 4 presents comparable data as 1–10 ppm of ammonia interacts with an aminated copper oxide treated *n*-type PS interface, converting this interface *in situ* to a copper oxynitride interface. Again, the nitridation of Cu*_x_*O decreases Lewis acidity and shifts the response of the modified nanostructures further to the soft acid side of Figure 2. It is tempting to hypothesize that the formation of the oxynitride should simply increase the basicity of the nanostructure surface and, thus, should decrease the response to NH_3_. However, this does not occur. The nitridated copper oxide is shifted further to the soft acid side of ammonia in Figure 2, dictating a greater HOMO-LUMO mismatch of interacting molecular orbitals. The IHSAB principle suggests, counter to intuition, that the response of the *in situ*-treated nitridated copper oxide interface should increase relative to that of Cu*_x_*O, precisely as is observed. In Figure 2, NO is positioned directly under the copper oxides. Nitridation shifts the copper oxides to the soft acid side and away from NO, leading to an increase in the HOMO-LUMO mismatch. As Figure 5 demonstrates, the reversible response of the oxynitride to NO increases relative to that of the Cu_X_O-decorated PS interface. The results in Figure 3, Figure 4 and Figure 5 strongly suggest that the IHSAB principle can be used as an important distinguishing principle of sensor response and the transformation from electron transduction to chemisorption. In support of this argument, initial results obtained for the nitridation of NiO lead to a decrease in response for NO; however, as would be predicted by the IHSAB model, the reversible response for interaction with NH_3_ increases [16]. The simplicity of the *in situ* nitridation process [10,16,18,19,20] can provide an important means of enhancing interface modification and selection.

The concept of *in situ* nitridation has now been extended. Initial results have been obtained for interfaces in which metal oxide nanostructures are functionalized with sulfur to form the oxysulfides. *In situ* treatment with R_2_S sulfides lowers the Lewis acidity of the treated metal oxide site (M^+*x*^) relative to the oxide and appears to convert the oxides to oxysulfides in analogy to the nitridation process. The substitution of sulfur for oxygen in the MO_2_ lattice would be expected to place more electron density on the M site and, hence, lower the Lewis acidity of M. In addition, an S in the lattice is softer and would have less negative charge. However, when the metal oxide interfaces are treated with the thiols, RSH, we observe the manifestation of an increase in the acidity, which suggests that there are thiol (SH) groups on the surface. In other words, we find evidence for an increase in the Brønsted acidity of the surface. The thiols are more acidic than alcohols, due to their weaker S-H bonds and the better ability of sulfur to hold the negative charge after proton loss.

**Figure 6:**
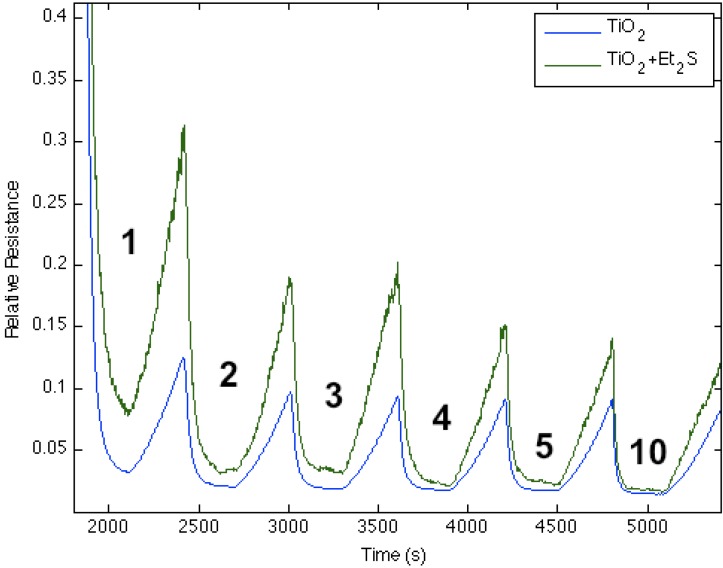
Response of diethyl sulfide, Et_2_S, -treated TiO_2_-deposited porous silicon (PS) interface to NH_3_. Exposure to TiO_2_-treated PS interface (**blue line**). Response of diethyl sulfide-treated TiO_2_ nanostructure-deposited PS interface to NH_3_ (**green line**). The Et_2_S-treated TiO_2_ deposited interface is made more basic relative to the PS and TiO_2_-treated PS acidic sites.

**Figure 7:**
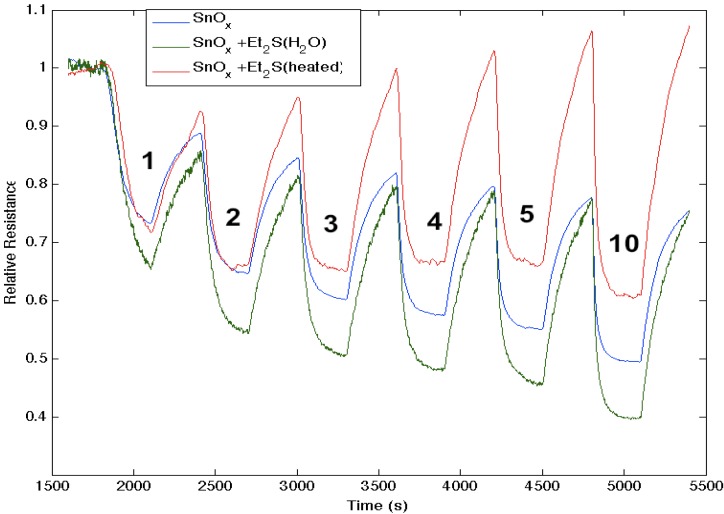
Response of diethyl sulfide, Et_2_S, -treated tin oxide-deposited porous silicon (PS) interface to NH_3_. Exposure to SnO*_x_*-treated PS interface (**blue line**). Initial exposure to Et_2_S with water present (**green line**). Response of diethyl sulfide-treated tin oxide nanostructure-deposited PS interface to NH_3_ after gentle heating to 80 °C to remove water (**red line**). The Et_2_S treated SnO*_x_*-deposited interface is a weaker Lewis acid relative to the PS and SnO*_x_*-treated PS acidic sites, where it is more acidic in the presence of water (See, also, the thiol results).

**Figure 8:**
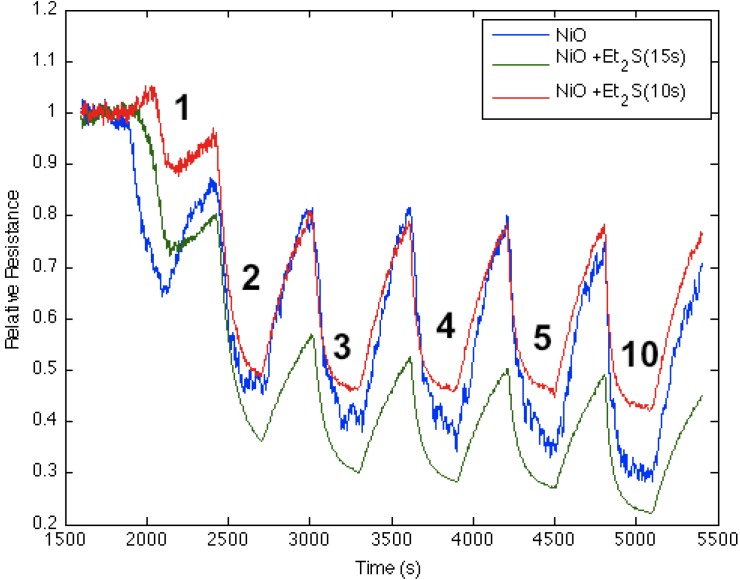
Response of diethyl sulfide-treated nickel oxide nanostructure-deposited porous silicon (PS) interface to NH_3_. Initial response of nickel oxide-treated PS (**blue line**), after treatment for 10 s with diethyl sulfide (**red line**), and after treatment for 15 s with diethyl sulfide (**green line**). The Et_2_S-treated NiO-deposited interface treated for 15 s is a weaker Lewis acid made more basic relative to the PS- and NiO-treated (Figure 1) PS interface.

**Figure 9:**
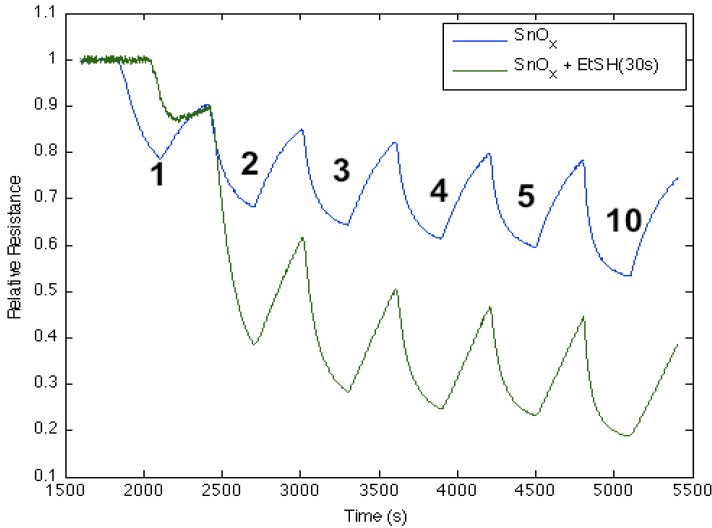
Response of ethanethiol-treated tin oxide nanostructure-deposited porous silicon (PS) interface to NH_3_ after exposure for 30 s (**green line**) *vs.* only tin oxide (**blue line**). The response of the thiol-treated SnO*_x_*-deposited interface is consistent with the introduction of S-H groups on the interface and an increased Brønsted acidity relative to the PS- and SnO*_x_*-treated PS acidic sites (Figure 2) after a 30 s exposure.

**Figure 10:**
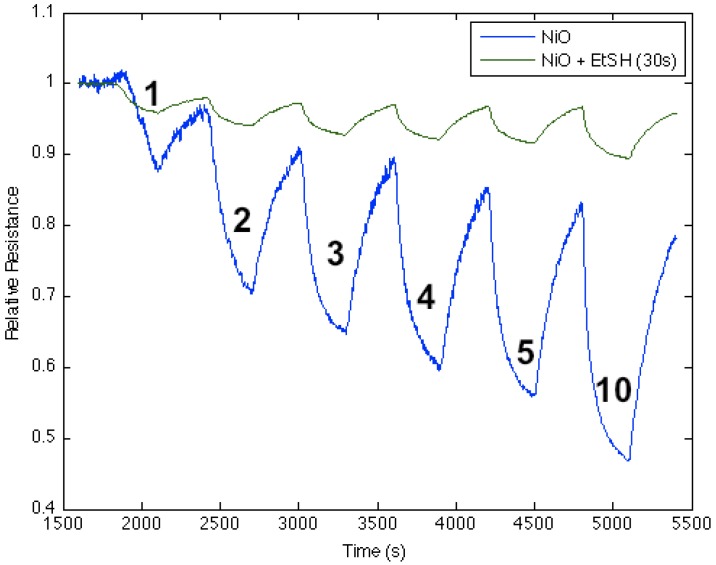
Response of ethanethiol-treated nickel oxide nanostructure-deposited porous silicon (PS) interface to NH_3_ exposure for 30 s (**green line**), and exposure only to nickel oxide (**blue line**). The thiol-treated NiO-deposited interface is more acidic than the NiO-treated PS acidic sites (Figure 1) after a 30 s exposure.

Figure 6, Figure 7, Figure 8, Figure 9 and Figure 10 demonstrate the results of the sulfur functionalization. Figure 6 corresponds to the responses observed when NH_3_ contributes electrons to a diethyl sulfide-treated titanium oxide-deposited PS interface. The process decreases the ability of the interface to extract electrons from NH_3_ as the majority charge carrier concentration (electrons) and the conductance for the diethyl sulfide-treated, TiO_2_-deposited PS interface decrease. Figure 2 suggests that a decreased Lewis acidity on treatment with Et_2_S produces a greater HOMO-LUMO orbital mismatch between TiO_2_ and NH_3_.

Figure 7 corresponds to the responses observed when NH_3_ contributes electrons to a diethyl sulfide, (C_2_H_5_)_2_S, treated tin oxide-deposited PS interface. After an initial treatment of the tin oxide-deposited surface, the diethyl sulfide treatment produces a significant increase in conductance relative to the surface deposited only with tin oxide. The process appears to increase the majority charge carrier concentration (electrons) and the conductance relative to an untreated PS interface for both the diethyl sulfide-treated and tin oxide-deposited PS interfaces [17]; however, mild heating (~80 °C) of the sulfidated surface decreases the conductance relative to the treated metal oxide-deposited surface. The conductance is now found to decrease relative to the tin oxide-deposited interface. Based on observations after treatment with EtSH (ethyl thiol) that we will outline, we suggest that the decrease of conductance is likely due to the removal of interacting water previously hydrating the sulfide. The sulfide in the absence of significant water interacts with the SnO*_x_* nanostructure-decorated interface to produce a site of decreased Lewis acidity, diminishing the HOMO-LUMO orbital mismatch with NH_3_. This is consistent with the diminished transfer of electrons from NH_3_ to the sulfur-substituted SnO*_x_*-treated interface. In contrast, Figure 7 demonstrates that the sulfidated surface can be hydrated and that this is manifest by an increase in the acidity of the surface sites relative to SnO*_x_*.

Figure 8 corresponds to the responses observed when NH_3_ contributes electrons to a diethyl sulfide-treated nickel oxide-deposited PS interface. The process increases the majority charge carrier concentration (electrons) and the conductance for both the diethyl sulfide-treated and nickel oxide-deposited (Figure 8, blue line) PS interfaces. The initial treatment of the nickel oxide-deposited surface with diethyl sulfide for ten seconds produces a decrease in conductance relative to the surface deposited only with nickel oxide (Note also Figure 1). However, red line in Figure 8 demonstrates that an increase to a 15 s exposure of the diethyl sulfide results in an increase in the conductance of the sulfur-treated surface relative to the metal oxide-deposited surface. Thus, the level of diethyl sulfide exposure must be carefully assessed and optimized [1,2,3,4,5,6]. Figure 2 suggests that the decreased Lewis acidity, which treatment with Et_2_S produces (substitution of S for O), will lead to a greater HOMO-LUMO orbital mismatch between NH_3_ and the nickel oxysulfide. This promotes a stronger reversible electron transduction with NH_3_ and, thus, an enhanced conductance. Figure 8 also suggests that it is possible to tune the Lewis acidity of the metal site in the oxide interface through controlled exposure to the sulfide.

In contrast to the sulfides, the thiols, RSH, are acidic rather than basic, and we find that their interaction with SnO*_x_*- and NiO-nanostructured oxide surfaces is the reverse of that for the sulfides. This suggests the presence of S-H bonds on the surface of the thiol-treated interface. S-H bonds on the surface of the interface emanating from the thiol can create Brønsted acid sites, which are manifest as the increased acidity of the doped metal oxide site.

Figure 9 corresponds to the responses observed when NH_3_ contributes electrons to an ethanethiol, CH_3_CH_2_SH, -treated tin oxide-deposited PS interface. The process increases the majority charge carrier concentration (electrons) and the conductance for both the ethanethiol-treated and tin oxide-deposited PS interfaces. Once optimized, the ethanethiol-treated surface displays a significant increase in conductance relative to the surface that is deposited only with tin oxide. This is consistent with the interaction of an acidic thiol that interacts with the SnO*_x_* nanostructure to produce a modification to the hard acid side of Figure 2. This process can occur if S-H bonds are formed on the interface surface providing a Brønsted acidity. This can create an effect similar to an increased HOMO-LUMO gap, due an increase in Lewis acidity. Additional data also suggests, in complement to the sulfides, that the degree of acidity of the SnO*_x_*-deposited surface can be varied, increasing the magnitude of conductance, in a controlled manner.

Figure 10 corresponds to the response observed when NH_3_ contributes electrons to an ethanethiol-treated nickel oxide-deposited PS interface. The process results in a decrease in the conductance relative to the nickel oxide-treated surface, as it decreases the majority charge carrier concentration (electrons) and the conductance relative to the NiO-deposited interface. The effect of the thiol suggests that it increases the acidity of the NiO-treated surface as it forms Brønsted acid S-H sites. Thus, the NH_3_- and thiol-treated interfaces are more closely matched with a decreased HOMO-LUMO gap separation.

The initial results, which we outline, suggest the possibility of a novel, general and readily applied approach to the formation of sulfur-functionalized interfaces, which may find application in the manipulation of biomolecules [21,22], for example, by simplifying the application of DNA oligonucleotides, that are now thiol-tagged, for surface immobilization [22]. In concert with those results obtained for nitridation, these studies also suggest the potential for extension to the remaining pnictogens, chalcogens and oxyhalides.

## 3. Experimental Section

Earlier, we described [9] highly efficient nanostructure modified interfaces on *n*-type PS, as we generated a micro-/nano-porous interface [1]. A hybrid etch procedure is used to generate the nanopore covered micropores depicted in Figure S1 [1,2,23,24]. The PS interface is generated by electrochemical anodization of 1–20 ohm-cm, *n*-type (phosphorous doped) (100) silicon wafers (Wafer World) (Figure 1). The anodization of the *n*-type wafers [25,26] is done under topside illumination using a Blak-Ray mercury lamp. The silicon wafer is etched in a 1:1 solution of HF and ethanol at a current between 8 and 15 mA/cm [25,26,27,28,29]. The anodized *n*-type sample is placed in methanol for a short period, subsequently transferred to a dilute HF solution for a 30-min period and, then, washed again in methanol. This process creates a porous structure with pore diameters on the order of 0.5–0.7 µm and pore depths varying from 50 to 75 µm (Figure S2). The micropores provide a medium for Fickian diffusion to the surface nanoporous layer.

The PS hybrid arrays of nanopore covered micropores are tested at room temperature for their individual sensor response. The nature of this response is based on the application of the IHSAB acid/base principle. The selection of the nanostructures and the variable surface sensitivities that are produced as they form *in situ* metal oxide deposits introduces a distinct systematics of design. The approach is unique in that the nanostructures are deposited fractionally to the PS micropores, and this fractional deposition *does not* require any time-consuming self-assembly within the pores. This is not a coating technique or one that requires an exacting structural film arrangement, but is, in fact, a much simpler process [1,2,23,24]. The combination of distinctly different responses observed can be used as a basis to develop selectivity. Results obtained with nanostructured deposits generated from electroless tin, nickel and copper, as well as nano-titania are considered in this study.

All of the nanostructured metals deposited to the PS surface are readily oxidized to SnO*_x_* (*x* = 2,4), NiO and Cu*_x_*O (*x* = 1,2), as demonstrated by XPS measurements [3,30]. The initially introduced nano-titania (anatase) may be crystalline, although we cannot be certain of this crystallinity after deposition to the PS interface [18].

Triethylamine (TEA) is introduced to the nanostructured metal oxides by direct *in situ* treatment. The metal oxide treated surface is exposed to the TEA for 10 s. The treated interface is subsequently washed in methanol to remove excess TEA and allowed to age for approximately 24 h. Sulfur is introduced to the nanostructured metal oxides through direct *in situ* treatment with diethyl sulfide. The metal oxide-treated surface is exposed to diethyl sulfide for 10 s. The thiols are introduced to the nanostructured metal oxides through direct *in situ* treatment with ethane thiol or butane thiol. The metal oxide-treated surface is exposed to the thiols for 30 s. The treated interface is subsequently washed in methanol to remove excess sulfide or thiol and allowed to age for approximately 24 h.

The sensors are evaluated in an unsaturated mode, since the time scale for reversibility may become an issue in a long-term saturated mode, and the longer term exposures are not necessary [1,2,23,24]. NH_3_ was pulsed onto these interfaces with a 300 s half-cycle followed by a 300 s half-cycle nitrogen cleaning. The numbers denote ppm exposure to NH_3_. The system was purged with ultrahigh purity (UHP) nitrogen for 1800 s before operation. The sensor response and recovery times for “sticky gases”, such as ammonia, are distinctly different, and full time recovery from the gas exposure takes longer than 300 s. This is the exposure time duration in the present configuration (Figure 2, [4]). However, the onset of the sensor response for these atmospheric pressure “open inlet” studies remains clearly visible. The behavior, which looks very much like the reverse of Figure 2 in [4] suggests that the responses for NH_3_ on PS are that of a gas whose interaction may be dominated by physisorption, but which also displays weak chemisorption. Purging the sensor surface with UHP N_2_ for longer durations improves the gradual shift to the initial base line. The return to baseline can also be further improved by more tightly constraining the gas flow path to the sensor surface.

In all cases, the analyte gas being sensed is brought to the hybrid surface after entrainment at room temperature in UHP nitrogen (99.999%+ Matheson). The typical resistances for the base PS structures range between 300 and 10,000 ohms at room temperature. The gas flow for the analyte and the entraining UHP nitrogen is controlled by MKS type 1179A mass flow controllers (MKS Instruments Andover, MA, USA). The mass flow controllers used to control the analyte gas and the entraining nitrogen flow responded in less than 2 s. The diffusion time of the analyte gas to the sensors, which provides the longest system time constant, varies from four to five seconds for the lowest analyte concentrations, to an order of 1 to 2 s for concentrations greater than 2 ppm. These are the delay times for the observation of a signal, due to the analyte in the supply line. The sensors respond to the analyte gas on a time scale much less than two seconds. The change in resistance is measured in one-second intervals using a DC current. This voltage bias used in these experiments is 3 volts, to obtain an optimum signal-to-noise ratio. An NI DAQPad-6015 (National Instruments, Austin, TX, USA) is used for gathering data and supplying the DC current. Labview software is used to control the experiment and record the results. MATLAB is used in the analysis of the data.

## 4. Conclusions

The data that we have presented suggests that we can vary the acid/base properties of the metal oxide (metal centers) outlined in Figure 2 and the sensor scale, varying the metal positive charge by doping *in situ* with sulfur and nitrogen substituted from appropriate precursors. For a TiO_2_ nanoparticle site, the Ti is nominally in the +4 oxidation state. Sulfur and nitrogen will donate electron density into the metal if they are substituted for oxygen. This will shift the metal toward the softer acid side in the top portion of Figure 2. Thus, the interaction with the fixed analytes catalogued in the bottom of this figure will increase, and the sensor signal will decrease. However, the change in Lewis acidity does not shift the doped TiO_2_ further to the right than the fixed position of NH_3_, so the signal response for the NH_3_ and NO will both decrease and remain of the same sign. However, for a sensing metal site between two analytes, for example, Ni^2+^, the situation is modified. Ni^2+^ is approximately equidistant between NH_3_ and NO. As its Lewis acidity decreases, the signal from NO will decrease and that for NH_3_ will increase. These patterns provide a basis for increasing the breadth of sensitivity matrices.

There are different ways to control the size of the interaction of those molecules that are to be sensed with variably doped metal oxide-sensing interfaces. If the orbital orientation at the surface is not correctly configured, there can be little binding with the lone pairs of the incoming molecules. In addition, a combination of molecular and surface steric effects could also block the interaction at the surface by preventing orbital overlap. If the donor orbital energy (highest occupied molecular orbital, HOMO) is not well matched with the acceptor (lowest unoccupied molecular orbital, LUMO), then the interaction will be weak. As the HOMO(donor)-LUMO(acceptor) energy gap decreases, there can be more charge transfer between the molecule and the sensor interface, leading to a stronger Lewis acid-base interaction. For Lewis acid-base bonds, the donor retains the electron pair, a prototypical example being BH_3_NH_3_, with a B-N bond dissociation energy (BDE) of 26 kcal/mol [31]. At the other extreme is the interaction of an anion and a cation forming an ionic bond with a much large BDE. If the electrons are fully shared, leading to the formation of a covalent bond, this can also lead to a large BDE. The IHSAB is, in large part, based on controlling the size of the Lewis acid-base bond dissociation energy.

Our results, thus, by far display a clear quantitative dependence on concentration; however, they are based on qualitative inferences from measuring the sensing signals from the interactions of molecules interacting with surfaces via donor-acceptor interaction. We intend to obtain more detailed physical measurements on the structures of the surfaces and the energetics of these surfaces. Molecular data needed to address the orbital energy arguments is available in terms of molecular proton affinities, acidities and ionization potentials, but this data are not broadly available for surfaces [32,33]. While our measurements now provide semi-qualitative data about the doped metal oxide surface sites, further experiments will help to quantify that data.

We have indicated how the fractional deposition of metal oxide nanostructures can be used as a means of obtaining distinct sensor responses that show the potential for combination in an array-based format. Within the framework of integral nanostructured island sites, the behavior of the interfaces that are generated appears to be well represented by the newly developing IHSAB model. Here, we have begun to expand the versatility inherent to the metal oxides and the range of sensor response through *in situ* amination, converting to the more basic oxynitrides, or through *in situ* interaction with the basic sulfides or acidic thiols to produce the more basic oxysulfides or their corresponding hydrogen-functionalized acidic counterparts. It is significant that these results can be obtained with a simple *in situ* treatment at the nanoscale.

In considering the current mode of interface preparation, we will want to better understand the change in electronic character of the interface sensing system and its interaction with an analyte as the analyte injects or removes charge from the semiconductor interface. It is apparent from the data, considered in Figure 2, Figure 3, Figure 4, Figure 5, Figure 6, Figure 7, Figure 8, Figure 9 and Figure 10, in combination, that the observed interactions and the conductometric response of the developed interfaces represents much more than a simple acid/base interaction. We must be concerned with how the occupied bands within the extrinsic semiconductor change as an analyte interacts with the interface. How does the change in the bands occur? How do the surface-based molecular orbitals rearrange and combine, and how does this affect the electronic properties, including the bandgap?

We suggest that it is appropriate to expand the selective deposition and *in situ* modification of nanostructured materials to create inexpensive microfabricated sensor platforms and develop “*materials selection tables*” built on the IHSAB model. It is desirable to expand the range of interactions forming “*materials sensitivity matrices*” for a given analyte. This will also enhance the capacity to sense a wider range of analytes and their mixtures. These efforts will also be the subject of future studies.

## Supplementary Materials

Supplementary File 1
