# Supplementary material for: Nanostructure-Directed Chemical Sensing: The IHSAB Principle and the Effect of Nitrogen and Sulfur Functionalization on Metal Oxide Decorated Interface Response
Source: Nanomaterials (Basel). 2013 Aug 7;3(3):469–85. doi: 10.3390/nano3030469 (PMC5304653; doi:10.3390/nano3030469)
Supplement: Supplementary File 1 [file nanomaterials-03-00469-s001.docx]

**Supplementary Information**

The reversible, sensitive, and selective interaction of nanostructures to Lewis basic analytes depends on their Lewis acid strength. This is summarized for select gases in Table S1 [1–6]. Materials including nanotitania > SnO_2_ > nanoalumina > NiO > Cu*_x_*O > Au*_x_*O (where the progression is from a strong to weak acid) have already been demonstrated for the detection of gases including NH_3_ [1,6], PH_3_ [5], CO [1,4], NO*_x_* [1,8], H_2_S [1,3], and SO_2_ [1,3] at the sub-ppm level. NH_3_ is a hard base. Its position among the bases is indicated in Table S1 which summarizes primarily the acid-base strength of ions associated with the oxides and a selection of molecules. From this table, we can infer the reversible interaction of NH_3_ with a variety of surfaces using the IHSAB model.

**Table S1.** Hard and soft acids and bases. Reproduced from [2], with permission from John Wiley and Sons-VCH.

| **Hard** | **Borderline** | **Soft** |
| --- | --- | --- |
| Acids H^+^, Li^+^, Na^+^, K^+^ | Fe^2+^, Co^2+^, Ni^2+^ | Cu^+^, Au^+^, Ag^+^, TI^+^, Hg^+^ |
| Be^2+^, Mg^2+^, Ca^2+^ | Cu^2+^, Zn^2+^, Pb^2+^ | Pd^2+^, Cd^2+^, Pt^2+^, Hg^2+^ |
| Cr^2+^, Cr^3+^, Al^3+^ | BBr_3_, Sn^+2^, NO_2_ | BH_3_, NO |
| SO_3_, BF_3_, Sn^+4^, Ti^+4^ |  |  |
| Bases F^−^, OH^−^, H_2_O, NH_3_ | NO_2_, SO_3_^2−^, Br^−^, H^−^, R^−^, CN^−^, CO, I |  |
| CO_3_^2−^, NO_3_^−^, O^2−^ | N_3_^−^, N_2_, H_2_S, SO_2_ | SCN^−^, R_3_P, C_6_H_5_ |
| SO_4_^2−^, PO_4_^3−^, ClO_4_^−^ | C_6_H_5_N, SCN | R_2_S, NO |

*The Interface*

The basic semiconductor interface used in this study has been discussed previously and is illustrated in Figure S1. This structure is produced by a hybrid etch procedure used to create the desired interfacial porous silicon support structure. The nanopore-covered microporous structure of the interface has been created specifically to facilitate efficient gaseous diffusion (Fickian) to the highly active nanostructure (red) modified nanoporous (green) coating. The surface-attached nanoparticles possess unique size-dependent and electronic structure properties that form a basis for changing the sensitivity for exposure to specific gases. This exposure alters the conductivity of the porous silicon (measured by microprobe circuitry) attached to the gold contacts shown in Figure 1. When operated in the electron transduction mode, the transfer of electrons to an *n*-type PS interface, as would occur with a basic analyte, increases the majority of charge carriers, which are electrons, decreases the conductometric resistance and increases conductance. The removal of electrons, as would occur with an acidic analyte, decreases the majority of charge carriers and the conductance and increases resistance. The opposite behavior will be observed for a *p*-type semiconductor interface.

A schematic of the etch procedure and the typical pore structure with pore diameters on the order of 0.5 to 0.7 µm and pore depths varying from 50 to 75 µm is shown in Figure S2.

**Figure S1.** Schematic representation of PS sensor system.


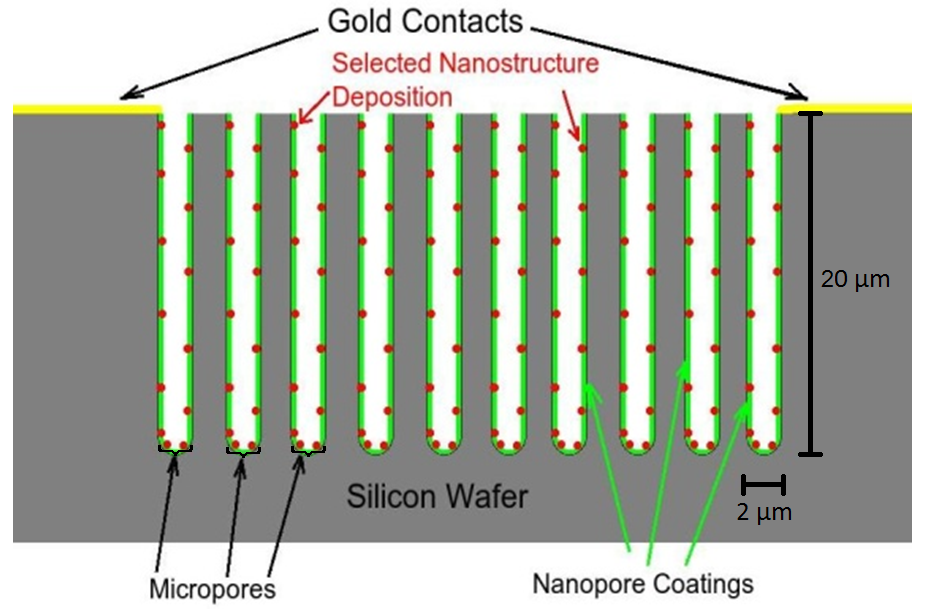


**Figure S2.** (**A**) Schematic view of apparatus used to generate the micro/nanoporous structure in *n*-type silicon. (**B**) Top and (**C**) side view of the pores. Reproduced from [2], with permission from John Wiley and Sons-VCH.


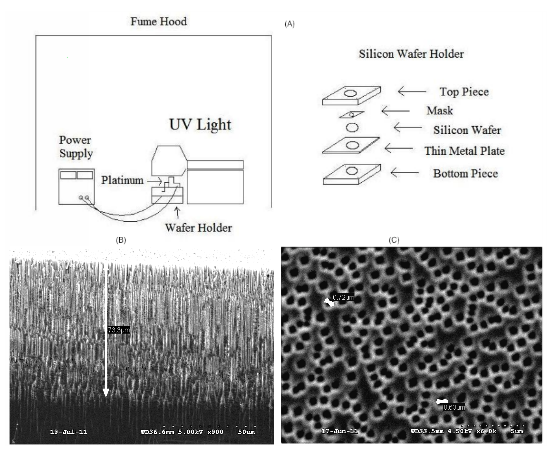


References are as listed in the main text.

© 2013 by the authors; licensee MDPI, Basel, Switzerland. This article is an open access article distributed under the terms and conditions of the Creative Commons Attribution license (http://creativecommons.org/licenses/by/3.0/).
